# Regional Changes in Cerebral Oxygenation During Repeated Passive Movement Measured by Functional Near-infrared Spectroscopy

**DOI:** 10.3389/fnhum.2015.00641

**Published:** 2015-11-25

**Authors:** Kazuhiro Sugawara, Hideaki Onishi, Atsuhiro Tsubaki, Haruna Takai, Yuta Tokunaga, Hiroyuki Tamaki

**Affiliations:** ^1^Institute for Human Movement and Medical Sciences, Niigata University of Health and WelfareNiigata, Japan; ^2^Department of Rehabilitation, Marukawa HospitalToyama, Japan; ^3^Department of Rehabilitation, Niigata Rehabilitation HospitalNiigata, Japan

**Keywords:** functional near-infrared spectroscopy, repetitive passive movement, movement frequency, time-dependent changes in cerebral oxygenation, systemic hemodynamic changes

## Abstract

The aim of this study is to investigate the influence of passive movement repetition frequency at 1.5-Hz and 1-Hz on changes in cerebral oxygenation and assess the temporal properties of these changes using functional near-infrared spectroscopy (fNIRS). No significant differences in systemic hemodynamics were observed between resting and passive movement phases for either 1.5-Hz or 1-Hz trial. Changes in cortical oxygenation as measured by fNIRS in bilateral supplementary motor cortex (SMC), left primary motor cortex (M1), left primary somatosensory cortex (S1), and left posterior association area (PAA) during passive movement of the right index finger revealed greater cortical activity at only 1.5-Hz movement frequency. However, there were no significant differences in the time for peak oxyhemoglobin (oxyHb) among regions (bilateral SMC, 206.4 ± 14.4 s; left M1, 199.1 ± 14.8 s; left S1, 207.3 ± 9.4 s; left PAA, 219.1 ± 10.2 s). Therefore, our results that passive movement above a specific frequency may be required to elicit a changed in cerebral oxygenation, and the times of peak ΔoxyHb did not differ significantly among measured regions.

## Introduction

Passive movement of limb segments is widely used in neurorehabilitation for stroke, traumatic brain injury, and neurological disease. The therapeutic efficacy of passive movement depends on the activation of relevant cortical circuits to induce compensatory neuroplasticity (Takeuchi and Izumi, 2013). It is thus critical to assess the strength and spatiotemporal features of cortical activity evoked by various passive movement protocols.

Numerous studies have measured brain activity following passive movement using functional magnetic resonance imaging (fMRI), positron emission tomography (PET), and electroencephalography (EEG); such studies have revealed that afferent signals from cutaneous mechanoreceptors, muscle spindles, and joint receptors activate not only the primary somatosensory cortex (S1) but also the primary motor area (M1) and supplementary motor cortex (SMC) (Mima et al., 1996; Weiller et al., 1996; Alary et al., 1998; Reddy et al., 2001; Radovanovic et al., 2002; Terumitsu et al., 2009; Szameitat et al., 2012; Sulzer et al., 2013; Chang et al., 2014). For instance, Terumitsu et al. (2009) reported activation of areas 3a, 3b, 1, 2, and 4 in the contralateral hemisphere by passive finger movement using fMRI, and Mima et al. (1999) reported activation of bilateral SMC in addition to contralateral S1 and M1 in response to passive finger movement using PET. In addition, Onishi et al. (2013) reported posterior association area (PAA) activation following passive finger movement using MEG. In reality, fMRI and PET measured cortical activity after repetitive passive finger movement, whereas EEG and MEG measured them in a short period of time after a single passive movement and not measuring the time-dependent changes during repetitive passive movement. Thus, little is known regarding time-dependent changes in cortical activity during repetitive passive movement.

Functional near-infrared spectroscopy (fNIRS) allows for non-invasive monitoring of regional neural activity as reflected by changes in oxyhemoglobin (oxyHb) and deoxyhemoglobin (deoxyHb) due to neurovascular-metabolic coupling (Kleinschmidt et al., 1996; Miyai et al., 2001; Tanosaki et al., 2003; Fuster et al., 2005; Shibuya et al., 2008). Moreover, although the spatial resolution of fNIRS is inferior to fMRI, it can monitor time-dependent changes in cortical activity with excellent temporal resolution. Thus, fNIRS may be more suitable for measuring the temporal distribution of cortical activity during repetitive passive movement.

The spatiotemporal features of cortical activity depend on the frequency of afferent input. Sadato et al. (1997) found that regional cerebral blood flow (rCBF) in the sensorimotor cortex, as measured by PET, increased with the frequency of voluntary finger movement, and Ibáñez et al. (1995) reported markedly increased rCBF in response to median nerve stimulation of more than 2 Hz, but no measureable changes in response to low frequency stimulation of 0.2 and 1 Hz. Therefore, peripheral stimulation above a certain threshold frequency appears to be necessary for measureable hemodynamic changes. The aim of this study is to investigate the influence of passive movement repetition frequency on changes in cerebral oxygenation and assess the temporal properties of these changes using fNIRS. We hypothesize that repetitive passive movement at 1.5-Hz would increase cortical activity to a greater extent in bilateral SMC and contralateral M1, S1, and PAA than 1-Hz passive movement due to higher frequency sensory input from peripheral receptors. Gibson et al. (2006) performed the optical tomography measurements when they conducted passive movements of the right and left arms for infants. They reported that cerebral blood flow in the contralateral M1 changed when they let one side arm exercise passively. However, they suggested that motor cortex is strongly activated because some infants resist during passive movement. In addition, it was difficult to distinguish the activity of S1 from M1 by passive movement definitely because infants have a smaller head than adults. Chang et al. (2014) investigated changes in the cerebral blood flow during passive movement of the right fingers and reported that a significant increase in oxyHb and totalHb values was observed for both the left SM1 and SMA, and a significant increase in totalHb value was observed for the left PMC and PFC during passive movement of the right fingers. However, this report involved passive movement at a constant frequency and did not investigate changes in the cerebral blood flow according to the change in passive movement frequency. There are several previous studies that measured the brain activity during passive movement using PET or fMRI; however, little is known regarding time-dependent changes in cortical activity during repetitive passive movement. The aim of this study was to investigate the influence of passive movement repetition frequency on changes in cerebral oxygenation and to assess the temporal properties of these changes using fNIRS.

## Materials and methods

### Participants

Thirteen healthy volunteers (age range, 21–31 years; mean ± standard deviation, 22.8 ± 2.7 years; 11 right-handed and 2 left-handed) participated in this study. None of the participants had engaged in recreational drug use or used medications affecting the central nervous system. All participants provided written informed consent. The study conformed to the Declaration of Helsinki and the Code of Ethics of the World Medical Association and was approved by the ethics committee of Niigata University of Health and Welfare.

### Experimental procedures

#### Passive finger movement

**Figure 1:**
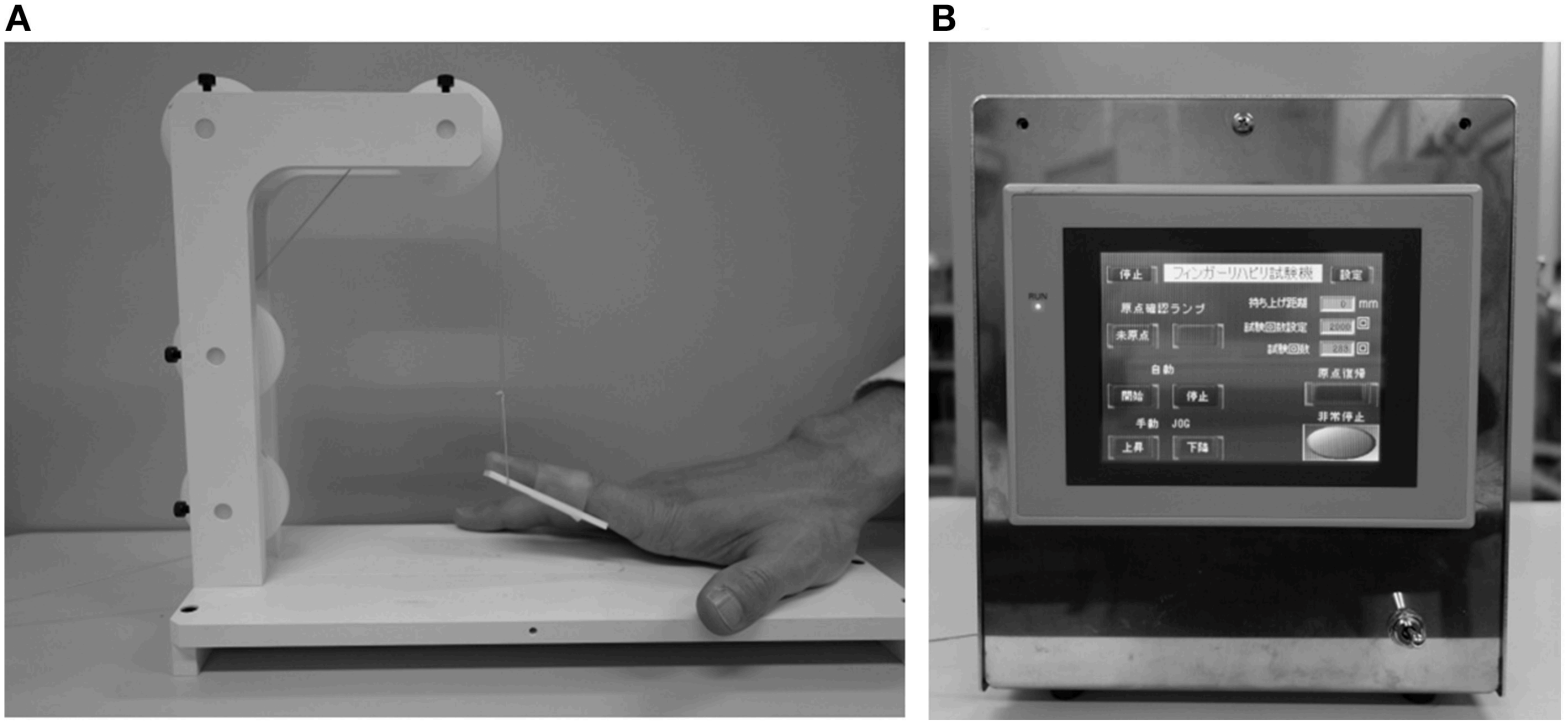
**(A)** Starting state of passive movement. **(B)** A custom-built device able to elevate the index finger with controlled velocity and frequency.

We used repeated passive movement of the right index finger to study changes in cortical oxygenation. The right forearm was placed comfortably on a table, with the elbow joint flexed at 70–80°. The forearm was in the pronated position, with all fingers and thumb extended naturally. Passive finger movements were generated by a custom-built device able to elevate the index finger with controlled velocity and frequency (Figures 1A,B). A plastic plate (1.5 cm height, 5.0 cm length, 2.0 mm thickness) was fixed to the planar side of the index finger from the proximal interphalangeal joint to the distal interphalangeal joint. A PE line (Super Strong; TORAY, Tokyo, Japan) attached to the plastic plate and a stepper motor were used to elevate the plate and passively extend the index finger. All subjects were instructed to relax their hands maximally and not to move the hand voluntarily or imagine such movements during passive extension.

**Figure 2:**
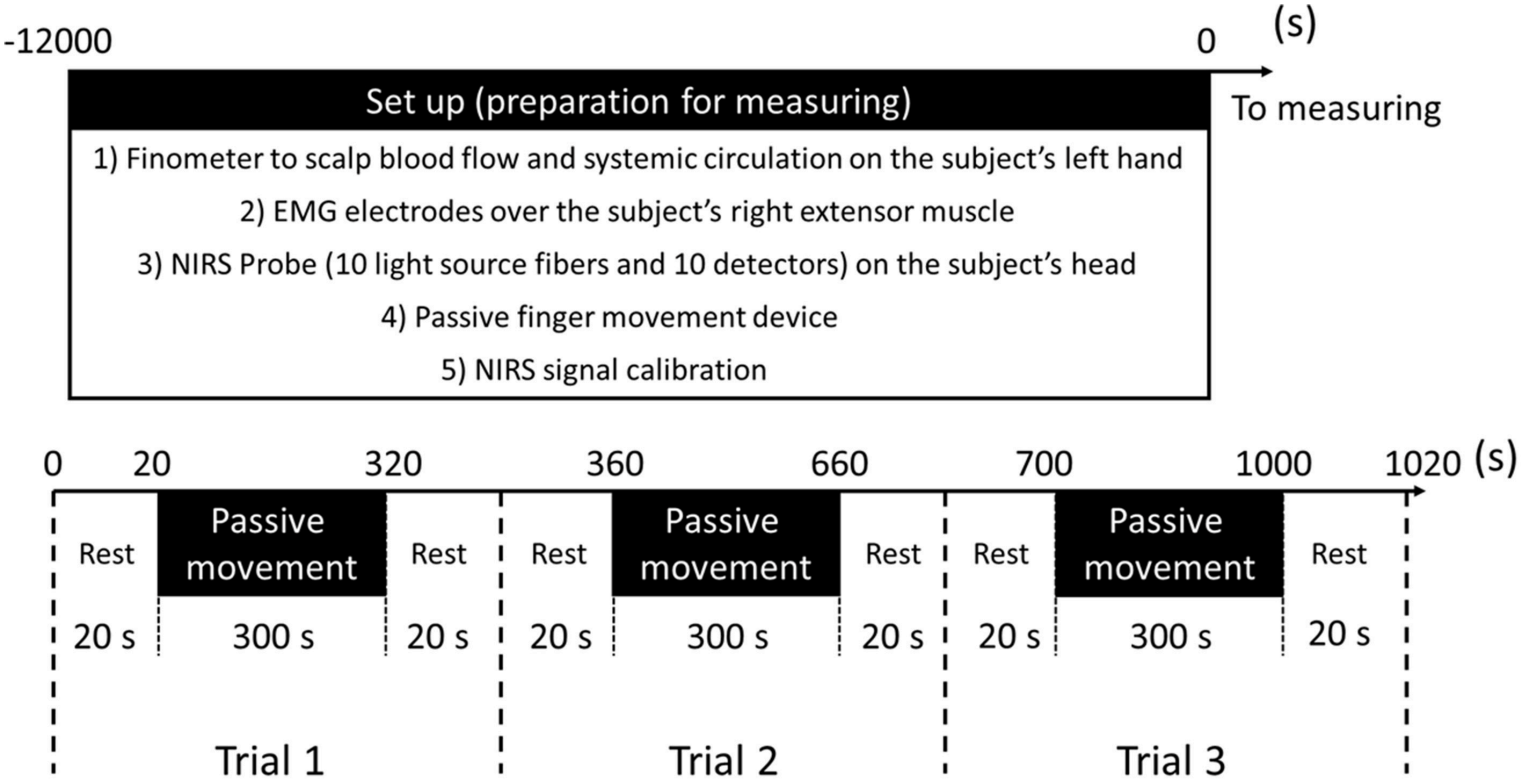
**Preparation for measurement and experimental protocol**.

To clarify the effects of passive movement frequency on cerebral hemodynamics, repeated finger extension was induced at 1.5-Hz and 1-Hz. In experiment 1 (11 subjects), passive finger movement was elicited at 1.5-Hz with approximately 40° of the MP joint extension and angular velocity of 241°/s (Onishi et al., 2013). In experiment 2 (11 subjects, including nine from experiment 1), passive finger movement was elicited at 1-Hz with the same angle and angular velocity. One trial comprised 20 s of rest, 300 s of passive movement, and another 20 s of rest. Each trial was repeated three times in a block. Thus, a single measurement lasted 1020 s (Figure 2).

### fNIRS

**Figure 3:**
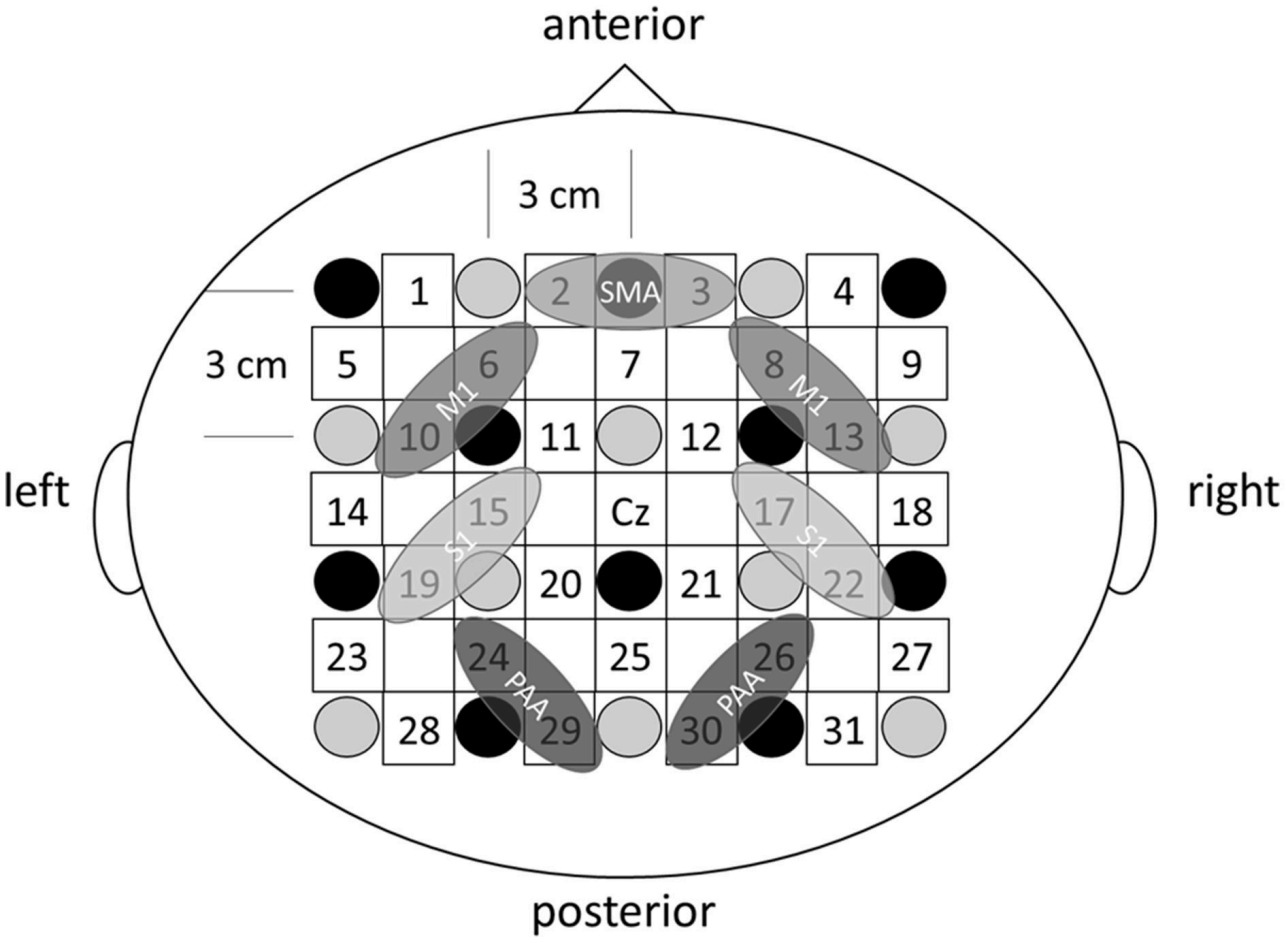
**The fNIRS probe set**. Black circles represent the optical illuminators, gray circles the optical detectors, and white squares the 31 channels.

The fNIRS system (OMM-3000; Shimadzu, Kyoto, Japan) comprised 10 light source fibers and 10 detectors forming 31 source-detector pairs (labeled channels 1–31, Figure 3) with interoptode distance of 3.0 cm. Each light source comprised three laser diodes with wavelengths of 780, 805, and 830 nm to detect changes in oxyHb, deoxyHb, and total hemoglobin (totalHb), respectively (Cope and Delpy, 1988). Signals were converted to concentrations (mM) by applying a modified Beer-Lambert law on line at a sampling rate of 160 ms. The Cz position of the international 10–20 system was used to ensure consistent optode placement between subjects. Since we aimed to examine cortical activity in both sensory- and motor-related area, the fNIRS array map covered the central and parietal areas of the scalp. The measured scalp area was divided into four regions of interest (ROIs) based on the functional anatomy of the medial frontal and medial parietal cortices (Miyai et al., 2001; Takeda et al., 2007; Shibuya et al., 2008; Chang et al., 2014). Changes in oxyHb, totalHb, and deoxyHb from rest were calculated during passive finger movement. Data from each cortical region are the mean values of all channels over that region. Left SI was covered by channels 15 and 19, right S1 by 17 and 22, left M1 by 6 and 10, right M1 by 8 and 13, bilateral SMC by 2 and 3, left PAA by 24 and 29, and right PAA by channels 26 and 30 (Figure 3). The baseline was calculated as the average signal during the 20 s rest period before the start of passive movement. Signals during passive movement were averaged over 10-s epochs (30 epochs during a movement period).

### Electromyography recordings

Electromyographic activity was also measured using a pair of Ag/AgCl electrodes (Blue-sensor NF-00; Ambu, Denmark) mounted over the right extensor muscle to detect any voluntary movements. Electromyography (EMG) signals (DL-140; 4 assist, Japan) were sampled at 1000 Hz (Power Lab; AD Instruments, CO), bandpass filtered at 0.5–500 Hz on line, and averaged over 20-s epochs.

### Blood pressure, heart rate, and skin blood flow measurements

fNIRS measurement of oxyHb reflects changes in not only cerebrocortical blood volume but also scalp blood flow and systemic circulation (Minati et al., 2011; Takahashi et al., 2011). Hence, we also measured Mean arterial pressure (MAP), heart rate (HR), and skin blood flow (SBF) during repetitive passive movement. MAP, HR, and SBF were also continuously monitored during repetitive passive movement tasks using a fingertip photoplethysmograph (Finometer MIDI; Monte System Corporation) on the left hand. Values were averaged over 10-s epochs.

### Data analysis

All data are presented as mean ± standard error of the mean. To assess changes in oxygenation over time, a repeated measures One-way analysis of variance (ANOVA) was used to test for significant effects of time on ΔoxyHb, ΔdeoxyHb, and ΔtotalHb in bilateral SMC, M1, S1, and PAA as well as for changes in MAP, HR, and SBF. *Post-hoc* analyses with Dunnett's correction were used for multiple comparisons. One-way ANOVA was used to compare difference in time of peak ΔoxyHb among scalp regions. *P* < 0.05 was considered significant.

## Results

**Figure 4:**
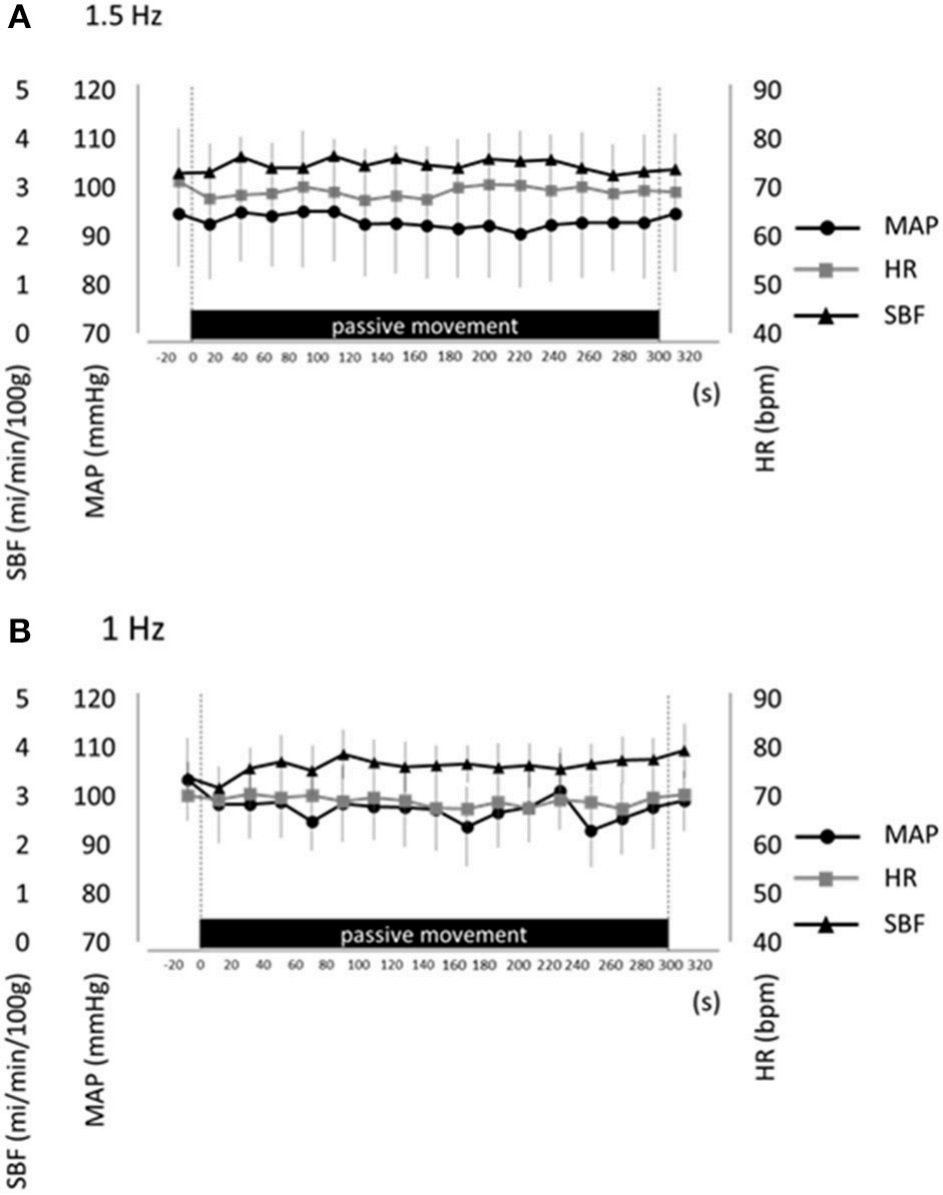
**Average mean arterial pressure (MAP, black circles), heart rate (HR, gray squares), and skin blood flow (SBF, black triangles) at rest and during repetitive passive movement at 1.5-Hz (A) and 1-Hz (B)**. Presented as mean ± standard error of the mean. There were no significant changes in these parameters during repetitive passive movement at both 1.5-Hz and 1-Hz.

To control potential influences of systemic hemodynamic changes and muscle contraction on passive finger movement-induced cortical activation, we measured MAP, HR, SBF, and finger EMG during both movement protocols (1.5 and 1-Hz). No significant differences in MAP, HR, and SBF were observed between resting and passive movement phases for either 1.5-Hz or 1-Hz trial (*P*>0.05; Figure 4). Moreover, EMG measures during passive finger movement showed no changes compared with the resting phase during trials, indicating no significant muscle contraction during passive movement.

**Figure 5:**
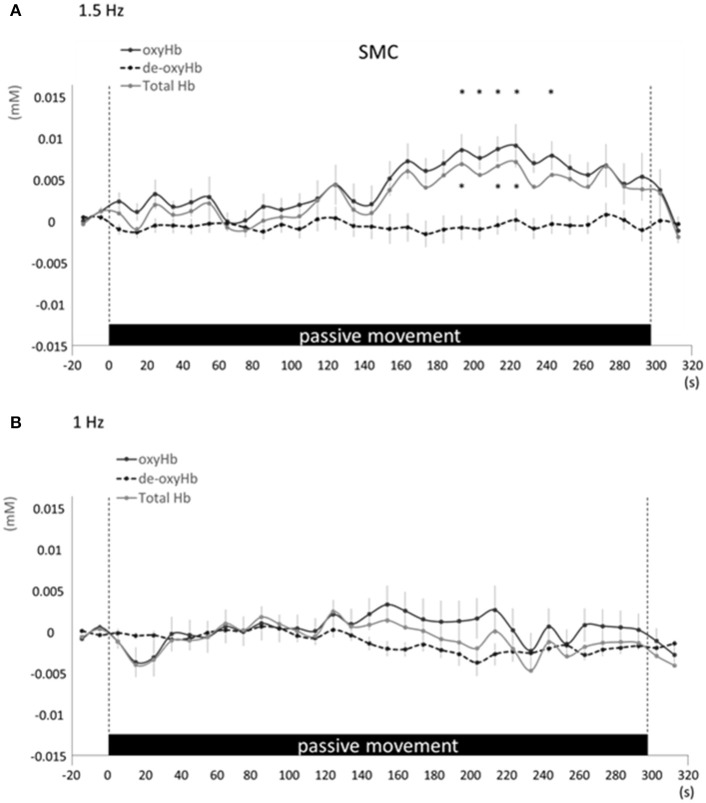
**Average oxyHb, deoxyHb, and totalHb in bilateral SMA at rest and during repetitive passive movement at 1.5-Hz (A) and 1-Hz (B)**. Black solid lines indicate oxyHb, black dotted lines deoxyHb, and gray solid lines totalHb.

**Figure 6:**
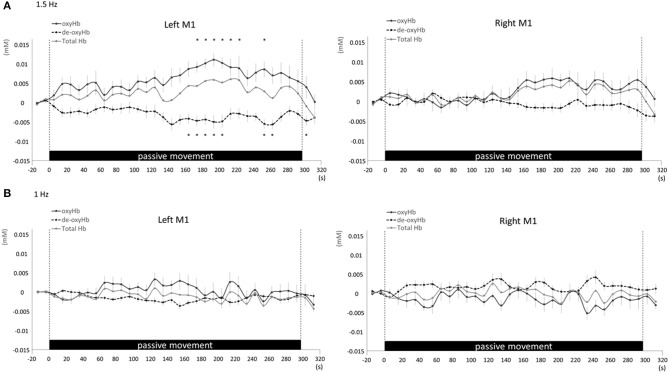
**Average oxyHb, deoxyHb, and totalHb in left and right M1 at rest and during repetitive passive movement at 1.5-Hz (A) and 1-Hz (B)**. Black solid lines indicate oxyHb, black dotted lines deoxyHb, and gray solid lines totalHb.

**Figure 7:**
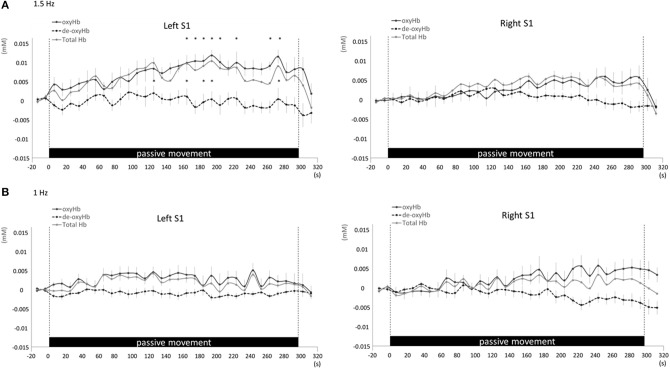
**Average oxyHb, deoxyHb, and totalHb in left and right S1 at rest and during repetitive passive movement at 1.5-Hz (A) and 1-Hz (B)**. Black solid lines indicate oxyHb, black dotted lines deoxyHb, and gray solid lines totalHb.

**Figure 8:**
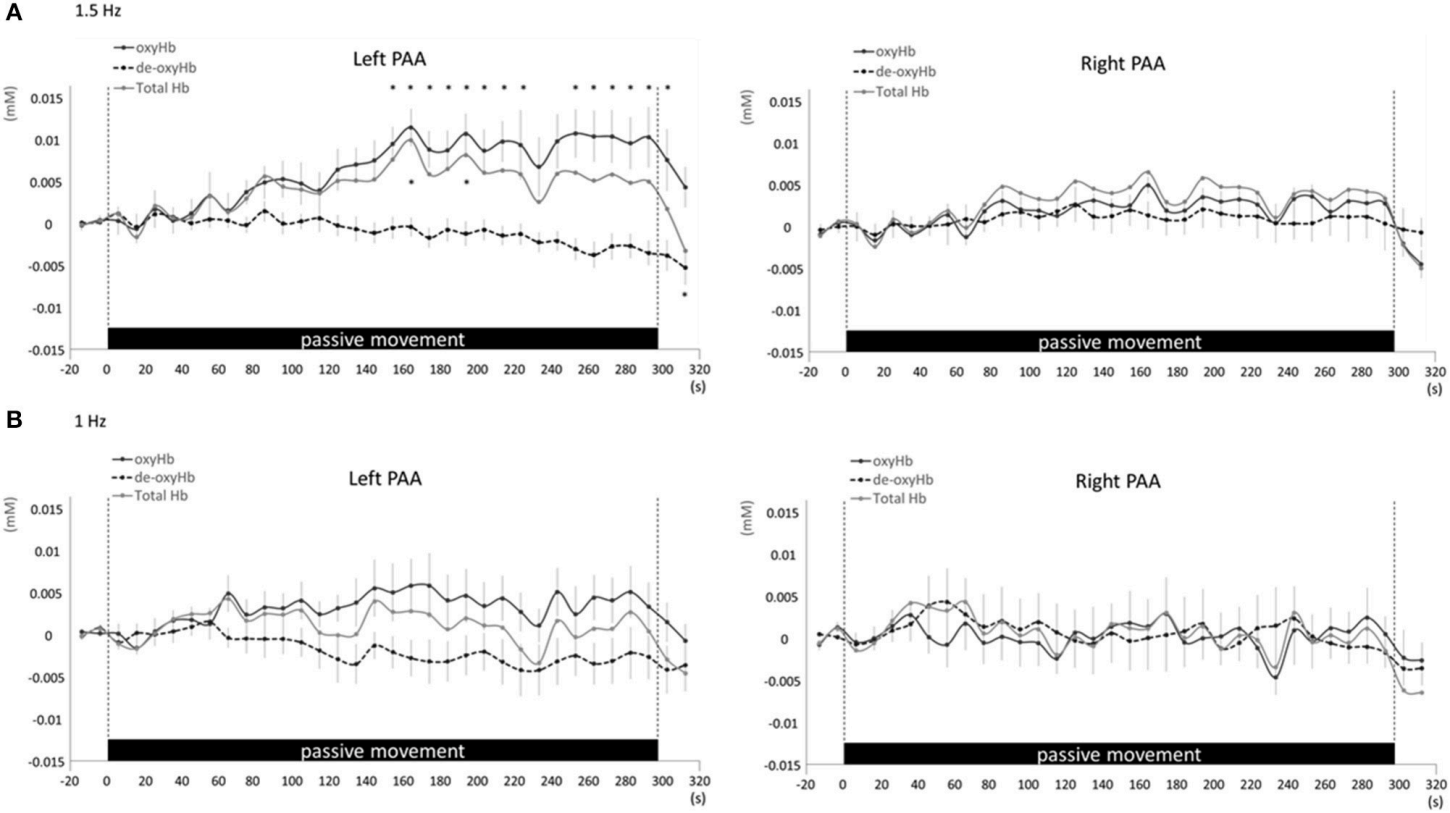
**Average oxyHb, deoxyHb, and totalHb in left and right PAA at rest and during repetitive passive movement at 1.5-Hz (A) and 1-Hz (B)**. Black solid lines indicate oxyHb, black dotted lines deoxyHb, and gray solid lines totalHb.

Changes in cortical oxygenation as measured by fNIRS in bilateral SMC, left M1, left S1, and left PAA during passive movement of the right index finger revealed greater cortical activity at the higher movement frequency (1.5-Hz vs. 1-Hz). Table 1 shows summary of the results of all regions at both stimulus frequencies. In bilateral SMC (Figure 5A), both oxyHb and totalHb significantly increased during repetitive passive movement at 1.5-Hz compared with the baseline (oxyHb, *F* = 4.144, *P* = 0.000; totalHb, *F* = 2.057, *P* = 0.000). Compared with the baseline, oxyHb increased significantly from 190 to 240 s (190 s, 0.00741 mM; 200 s, 0.00706 mM; 210 s, 0.00745 mM; 220 s, 0.00764 mM; and 240 s, 0.00631 mM) (*P* < 0.05) and totalHb increased from 190 to 220 s after the starting passive movement (190 s, 0.00659 mM; 200 s, 0.00648 mM; and 220 s, 0.00691 mM) (*P* < 0.05). No significant changes in deoxyHb were observed in SMC during repetitive passive movement at 1.5 Hz (*P* > 0.05). In contrast, no significant changes were observed in response to 1-Hz passive finger movement in the bilateral SMC (Figure 5B). In left M1 (Figure 6A), oxyHb also increased and deoxyHb decreased significantly during repetitive passive movement at 1.5 Hz (oxyHb, *F* = 2.591, *P* = 0.000; deoxyHb, *F* = 2.122, *P* = 0.000). Compared with the baseline, oxyHb increased significantly from 170 to 260 s (170 s, 0.00934 mM; 180 s, 0.00980 mM; 190 s, 0.01063 mM; 200 s, 0.01042 mM; 210 s, 0.0972 mM; 220 s, 0.00976 mM; and 250 s, 0.00855 mM) (*P* < 0.05) and deoxyHb decreased from 160 to 300 s after the starting passive movement (160 s, −0.00505 mM; 170 s, −0.00404 mM; 180 s, −0.00478 mM; 190 s, −0.00485 mM; 200 s, −0.00448 mM; 250 s, −0.00537 mM; 260 s, −0.00547 mM; and 300 s, −0.00371 mM) (*P* < 0.05). No significant changes in totalHb were observed in left M1 during repetitive passive movement at 1.5 Hz (*P* > 0.05). In contrast, no significant changes in oxygenation were observed in right M1 during repetitive passive movement at 1.5-Hz (*P* > 0.05). No significant changes were observed in both right and left M1 at 1-Hz (*P* > 0.05; Figure 6B). Similarly, signals were restricted to the contralateral side and were frequency dependent in S1 and PAA. In left S1 (Figure 7A), oxyHb and totalHb increased significantly during repetitive passive movement at 1.5-Hz (oxyHb, *F* = 2.561, *P* = 0.000; totalHb, *F* = 2.156, *P* = 0.000). Compared with the baseline, oxyHb increased significantly from 160 to 270 s (160 s, 0.00870 mM, 170 s, 0.0091 mM; 180 s, 0.00940 mM; 190 s, 0.01060 mM; 200 s, 0.00901 mM; 220 s, 0.00810 mM; 260 s, 0.00801 mM; 270 s, and 0.0102 mM) (*P* < 0.05) and totalHb increased at 120 s (0.0102 mM), 160 s (0.00823 mM), 180 s (0.00718 mM), 190 s (0.00845 mM), and 270 s (0.00798 mM) after the starting passive movement (*P* < 0.05). No significant changes in deoxyHb were observed in left S1 during repetitive passive movement at 1.5 Hz (*P* > 0.05). No significant change in oxygenation was observed in right S1 during passive movement at 1.5-Hz (*P* > 0.05; Figure 7A). No significant changes were observed in both right and left S1 at 1-Hz (*P* > 0.05; Figure 7B). In left PAA (Figure 8A), oxyHb, totalHb, and deoxyHb changed significantly during repetitive passive movement at 1.5-Hz (oxyHb, *F* = 4.859, *P* = 0.000; totalHb, *F* = 2.469, *P* = 0.000; deoxyHb, *F* = 2.520, *P* = 0.000). Compared to baseline, oxyHb increased significantly from 150 to 300 s (150 s, 0.00812 mM; 160 s, 0.01011 mM; 170 s, 0.0807 mM; 180 s, 0.00804 mM; 190 s, 0.00904 mM; 200 s, 0.00806 mM; 210 s, 0.00871 mM; 220 s, 0.00864 mM; 250 s, 0.00907 mM; 260 s, 0.00897 mM; 270 s, 0.00900 mM; 280 s, 0.00809 mM; 290 s, 0.00903 mM; and 300 s, 0.00701 mM) (*P* < 0.05) and totalHb from 160 s (0.00620 mM), 190 s (0.00731 mM), whereas deoxyHb decreased at 310 s (−0.00489 mM) after the start of passive movement (*P* < 0.05). No significant changes in oxygenation were observed in right PAA during repetitive passive movement at 1.5-Hz (*P* > 0.05). Furthermore, there were no significant changes in both right and left PAA oxygenation during repetitive passive movement in response to 1-Hz movement (*P* > 0.05; Figure 8B).

**Table 1:** **Summary of the results of all regions at both stimulus frequencies**.

	**SMC**	**left M1**	**Right M1**	**Left S1**	**Right S1**	**Left PPC**	**Right PAA**
**1.5 Hz**							
oxyHb	+	+	−	+	−	+	−
deoxyHb	−	+	−	−	−	−	−
totalHb	+	−	−	+	−	+	−
**1 Hz**							
oxyHb	−	−	−	−	−	−	−
deoxyHb	−	−	−	−	−	−	−
totalHb	−	−	−	−	−	−	−

*+, Significant change compared with baseline; −, no significant change compared with baseline*.

There were no significant difference in the times of peak oxyHb in bilateral SMC, left M1, left S1, and left PAA during repetitive passive movement at 1.5-Hz among regions (bilateral SMC, 206.4 ± 14.4 s; left M1, 199.1 ± 14.8 s; left S1, 207.3 ± 9.4 s; left PAA, 219.1 ± 10.2 s) (*F* = 0.507, *P* = 0.688).

## Discussion

We investigated regional changes in cerebral oxygenation during repetitive passive movement at two movement frequencies using fNIRS. No changes in cerebral oxygenation were observed in bilateral SMC, left M1, left S1, or left PAA in response to 1-Hz movement of the right index finger, whereas all these regions exhibited significant changes in oxygenation during repetitive passive movement at 1.5-Hz. Thus, cortical activity in response to passive movement is dependent on movement frequency likely because of activation of muscle, ligament, and joint afferents (Xiang et al., 1997; Alary et al., 1998; Mima et al., 1999; Onishi et al., 2013; Chang et al., 2014). These results provide a foundation for investigating optimal passive movement patterns for targeted cortical activation in rehabilitation.

In both rats and humans, the magnitude of the change in somatosensory area rCBF after electrical stimulation is dependent on stimulus frequency (Ngai et al., 1999; Matsuura and Kanno, 2001; Tanosaki et al., 2003; Ureshi et al., 2004). Ibáñez et al. (1995) reported a progressive increase in somatosensory area rCBF as peripheral stimulation was increased from 2 to 4 Hz but no change from rest with frequencies of 0.2 and 1-Hz. Thus, the frequency of afferent input and corresponding degree of cortical excitation are positively correlated with rCBF. Afferent input from cutaneous, muscle, and ligament receptors was likely greater during 1.5-Hz passive movement than during 1-Hz passive movement, thereby inducing a greater regional metabolic demand in afferent projection regions of cortex, resulting in greater changes in tissue oxygenation. Therefore, passive movement above a specific frequency may be required to elicit a change in cerebral oxygenation.

Several previous human studies using various neuroimaging modalities have also reported activation of bilateral SMC and contralateral S1, M1, and PAA in response to passive hand movement (Xiang et al., 1997; Alary et al., 1998; Mima et al., 1999; Hori et al., 2006; Terumitsu et al., 2009; Onishi et al., 2013). We observed contralateral M1 and S1 activation during passive movement in accordance with numerous previous studies (Radovanovic et al., 2002; Terumitsu et al., 2009; Onishi et al., 2013; Sulzer et al., 2013; Chang et al., 2014). We also observed oxyHb changes in bilateral SMC. Mima et al. (1999) reported activation of contralateral M1, S1, and bilateral SMC after middle finger passive movement. Reddy et al. (2001) observed deficits in SMC activation as measured by fMRI during passive movement in patients with sensory neuropathy, suggesting that bilateral SMC receives somatosensory input from the thalamus and participates in feedback. The PAA, including area 5 and 7 in the posterior wall of the central sulcus, is considered a region of higher-level sensory processing than S1 (Kaas, 2004) because it receives both direct and polysynaptic projections from areas 3a, 2, and other somatosensory areas (Prevosto et al., 2011; Sato et al., 2012). Onishi et al. (2013) reported PAA activity at 90 ms following passive movement using MEG. Similarly, we observed the change of oxygenation in left PAA approximately 160 s after starting repetitive passive movement, indicative of higher order somatosensory processing in response to repetitive passive movement. MEG can be used to measure the change of magnetic fields associated with activities of neural sources (Onishi et al., 2013). The PAA activity reported in Onishi's study represented the peak latency, which was calculated by averaging the change in magnetic field following passive movement, of neural activities evoked by the passive movement. The current study reported that the change of oxygenation associated with the neural activities were measured using fNIRS. The measured changes of oxygenation and continuation time occurred by repetitive passive movement were significantly different from the previous study. Left M1 was the only region exhibiting a significant decrease in deoxyHb. Although oxyHb reliably reflects brain activation (Hoshi et al., 2001), regional deoxyHb is more affected by oxygenation of venous blood and blood volume, and it tends to change unpredictably with decreasing CBF (Toronov et al., 2000; Hoshi et al., 2001). Thus, the observed change may reflect the greater propensity for wide fluctuations in deoxyHb. However, Chang et al. (2014) also reported a significant deoxyHb decrease in contralateral sensorimotor cortex during repetitive passive movement, suggesting that this change is directly related to the task.

fNIRS measurement of oxyHb reflects changes in not only cerebrocortical blood volume but also scalp blood flow and systemic circulation (Minati et al., 2011; Takahashi et al., 2011). Hence, we also measured MAP, SBF, and HR during repetitive passive movement. Previous studies that recorded the scalp blood flow and systemic circulation as heart rate, the mean blood pressure during fNIRS measurement revealed that the regional and systemic changes in the cardiovascular state strongly influence fNIRS signal (Tachtsidis et al., 2008; Minati et al., 2011; Tsubaki et al., 2013). When we performed the task as passive movement without muscle contraction and change of limb positions, these measurements confirmed no changes in systemic hemodynamics at both passive movement frequencies. However, our results showed that oxyHb in the regions of SMC, left M1, left S1, and left PAA increased. Therefore, we believed that the changes in fNIRS observed during passive movement at 1.5 Hz showed the change in cerebral blood flow went with the cortical activity. These measurements confirmed no changes in systemic hemodynamics at both passive movement frequencies, strongly suggesting that the signals measured by fNIRS were due entirely to changes in cerebral cortex oxygenation.

A previous study (Chang et al., 2014) that investigated the changes in cerebral blood flow during passive movement reported the amount of change in oxyHb and totalHb to be maximum for the SM1 region at approximately 20 s after the initiation of passive movement; this amount of change corresponded to approximately 0.02 mM. In this study, change in oxyHb and totalHb was recognized at approximately 160 s after the initiation of passive movement; this amount of change corresponded to a value lower than that observed in the precedent study. We performed passive movement for only the index finger in this study; however, they performed passive movement for all fingers in the precedence study. Therefore, we suggested that the amount of afferent input increased and resulted in a larger change in cerebral blood flow compared with that observed in the precedent study.

Although cortical activity during repetitive passive movement has been measured in numerous studies, the modalities used have not been optimal for measuring time-dependent changes. EEG/MEG can offer good localization accuracy within a few millimeters; however, it is necessary to average on responses obtained from multiple peripheral stimuli and movement. Thus, it is difficult to catch sustained changes of cerebral activity during consecutive external stimuli and movement tasks. In addition, given the longer response time found in this study, fMRI is also able to capture the changes, though its time resolution is inferior to fNIRS. It is possible to measure cortical activities on a long-term during sustained stimuli because fNIRS used for this study has excellent temporal resolution. Therefore, fNIRS may be more suitable for measuring the temporal distribution of cortical activity during repetitive stimuli as in this study. From this study, it is clear that such changes occur, at least when the frequency of movement is above a certain threshold; however, the oxygenation change in the regions associated with passive movement necessitates approximately 160 s more, in this case at 1.5-Hz. However, further fNIRS measurements are warranted to examine the spatiotemporal characteristics of the cortical response over a broader frequency range.

### Conflict of interest statement

The authors declare that the research was conducted in the absence of any commercial or financial relationships that could be construed as a potential conflict of interest.
